# Solidifying the minority high school student pathway: evidence from the health professions recruitment and exposure program

**DOI:** 10.3389/fpubh.2024.1408859

**Published:** 2024-06-05

**Authors:** Murtala I. Affini, Ashley Diaz, Daniel J. Dolan, Symphony Fletcher, Isra Hasnain, Isaiah Selkridge, Stephanie Washington, Abdullah Pratt

**Affiliations:** ^1^Pritzker School of Medicine, University of Chicago, Chicago, IL, United States; ^2^Department of Emergency Medicine, The University of Chicago Medical Center, Chicago, IL, United States

**Keywords:** health care professionals, pipeline program, underrepresented minorities (URM), diversity and inclusion, medical education

## Abstract

**Purpose:**

The objective of this report is to provide longitudinal insights on the impact of a health professions exposure program for high school students of underrepresented backgrounds in medicine.

**Context:**

Medical students at the University of Chicago reviewed data from their chapter of the Health Profession Recruitment and Exposure Program (HPREP) from 2016–2021 to discover trends in enrollment. This data is documented in the context of the program's mission to increase awareness of health disparities, the success of prominent alumni, and recent community efforts post-COVID-19 pandemic.

**Findings:**

Two hundred and ninety-nine high school students participated in the University of Chicago HPREP program between 2016 and 2021. Participants ranged in age from 12 to 18 years (mean = 16, SD = 1) and 74% (*n* = 222) were female students. Of 252 respondents, 58% (*n* = 147) identified as Black or African American, 31% (*n* = 77) identified as Latinx or of Hispanic origin, and 10% (*n* = 24) identified as another race or as bi-racial. Ten or fewer black male students participated in the program every year, including the 2020–2021 year in which 61 students participated.

**Conclusions:**

HPREP has played an important role in shaping the face of health care, especially in Chicago. The data suggests significant increases in the number of underrepresented minority women becoming physicians and serving Chicago communities in the next decade. Pathway programs for underrepresented students in medicine should be strategic in recruiting and educating future health professionals based on future workforce needs.

## 1 Introduction

Diverse health care teams are associated with improved patient outcomes (1). There is no better time to address this topic. Currently, black patients are twice as likely as white patients to have negative identifiers in their health records and black maternal mortality rates are three times higher than the rate of their counterparts (2, 3). Achieving this diverse health care team becomes difficult when < 10% of physicians are people of color, even though 25% of the U.S. population represents those demographics (4, 5). Compared to the 47% increase in medical school applicants overall, medical school matriculants from ethnic and racial minority backgrounds have grown by < 2% since 1980 (6).

While more medical schools are being built to address the national doctor shortage, addressing representation has been more challenging. Black student representation amongst medical students decreased from 8.1% in 1994 to 7.1% in 2015 (7). A report from the Association of American Medical Colleges of medical school applicants from 1978 to 2014 found that the number of black men decreased, even though every other minority group increased representation during this time (8). Despite increased college graduation rates for black males and efforts to improve diversity in medical school applicants, the number of black male applicants in 1978 to 2014 dropped from 1,410 to 1,337; matriculants dropped from 542 to 515 during that same time interval (8, 9).

Underrepresented women in the medical field - while rising in number - have had difficulty reaching specific medical specialties and positions of power. Even though one study found that women constituted nearly 50% of the graduating medical student class in 2012, only 7 out of 20 of the largest medical specialties had women entering at proportions of 50% or more, with more underrepresentation seen in African American and Hispanic populations in particular (10). In addition, underrepresented in medicine (URM) women are marginalized in nearly all specialties in academic medicine professorship outside of lecturer and instructorship positions (11) (for program definition of URM, see Supplementary Appendix). Only 14% of United States academic department chairs were women, with non-White women consisting of only 3% of that number (12). Cited reasons for these discrepancies include workplace microaggressions that create a discriminatory environment, a lack of mentorship and opportunities for URM populations and women due to decreased visibility and leadership of minorities, and a culture of exclusion within hospitals' traditions that attack their sense of belonging (10).

Patterns of underrepresentation are present in other health-related fields, which play a role in provider-level decision making and biases. In 2000, over 2,250 veterinary degrees were awarded across the US, with only 74 people of Hispanic descent, 49 African Americans, and 14 Native-American receiving degrees (13). The U.S. Department of Health found that nursing, the nation's largest health care profession, was 4.6% African American, 1.8% Latinx, 0.4% Indigenous or Alaskan Native, and 1.5% from two or more racial backgrounds in a 2004 survey (14). 81% of nurses in 2020 were White/Caucasian (15). While not representative of the US population, the nursing population is becoming more racially diverse. Nursing has historically been a female-dominated practice, with 10% of the nursing workforce being male (16). Perhaps more problematic is that men in nursing have dominated managerial positions (17, 18). Across all health fields, women make up 78.4% of healthcare and social workers in the US, however, representation of women in individual fields like medicine, nursing and physical therapy is unequally distributed (19). Mirroring the importance of diverse physicians from underrepresented backgrounds in medicine, diversity in these fields may improve patient experience (17, 20).

Efforts to address these growing issues are limited so far, and notably lack long-term outcomes (21). Medical students at the University of Pennsylvania designed a free guide to the medical school admissions process to support underrepresented and/or low-income students (4). Other programs have been administration-led, like the Florida State University School of Medicine “Bridge to Clinical Medicine” program that aims to accept more diverse medical students (16). However, these types of initiatives are not designed to expose medicine to students from underrepresented backgrounds. Early health care exposure is known to have more influence in increasing interest in medicine compared to late intervention (22).

To help improve diversity among health care professionals, the Health Professions Recruitment and Exposure Program (HPREP) brings high school students from URM backgrounds to science-related activities as well as multiple health careers (23). This program also allows students to improve important skills such as working in teams, performing research, and public speaking (see Supplementary Appendix). The goal of this case study is to present insights from the University of Chicago chapter of HPREP during the 2016–2021 program years within the context of its community and national trends. This report also describes the positive impact similar initiatives can promote and encourages them to build upon the lessons learned from this case experience in the future.

## 2 Context

The University of Chicago Pritzker School of Medicine has housed a chapter of HPREP that allows Chicago youth to interact with medical students, physicians, nurses, physical therapists, pharmacists, scientists, and other health professionals based on the South Side of Chicago since 2004. This is performed by hosting 6 weekly sessions for roughly 7 h each week. In keeping this program thriving for 20 consecutive years, this chapter hopes to help cultivate the next generation of Black and Brown healthcare professions who will 1 day serve their local communities as providers.

HPREP focuses its recruitment to schools in Chicago's South and West sides. Recruitment consists of emailing, visiting partnered schools, and presenting the HPREP program in person to students. Bus passes are provided to help students transport from their communities to the University of Chicago Pritzker School of Medicine and back every Saturday. The program consists of in-person educational activities like suture practice, “Stop the Bleed” training, and presentations related to decreasing local health care disparities (see Supplementary Appendix). Post-workshop surveys are distributed to students to understand their thoughts on each activity. Post-program evaluation surveys are sent out during the final Saturday while students are still on campus. Food and drinks are catered through local stores and businesses. All associated costs are paid for through an annual budget allocated by the University of Chicago Office of Civic Engagement.

During the 2020–2021 “pandemic” season, program leadership reached out to school counselors individually to advertise the program to students (see Supplementary Appendix). Scheduling and operations that year were significantly altered to continue to engage prospective high school students. The program was virtual with Zoom sessions. Program leadership adapted the normally in-person activities to be suitably completed from home (see Supplementary Appendix). Project materials were mailed to residences, including diplomas upon completion of the program. Other mailed items included T-shirts and stethoscopes that were donated by the University of Chicago Pritzker School of Medicine Admissions team and the University of Chicago Department of Surgery.

## 3 Detail to understand key programmatic elements

Between 2016 and 2021, the University of Chicago HPREP chapter attracted 299 students from 61 total schools. Of these schools, ~5% were private and the rest were public, including charter and magnet schools. Twelve (20%) more student participated in the 2021 cohort compared to the 2020 cohort. The median distance of these schools from the UChicago main campus is 10.2 miles. HPREP has also recruited students from more than 20 organizations around Chicago with various missions to enhance research experience, communication skills, and college readiness among minority populations. The median distance of these organizations from the UChicago main campus is 8.3 miles.

**Figure 1:**
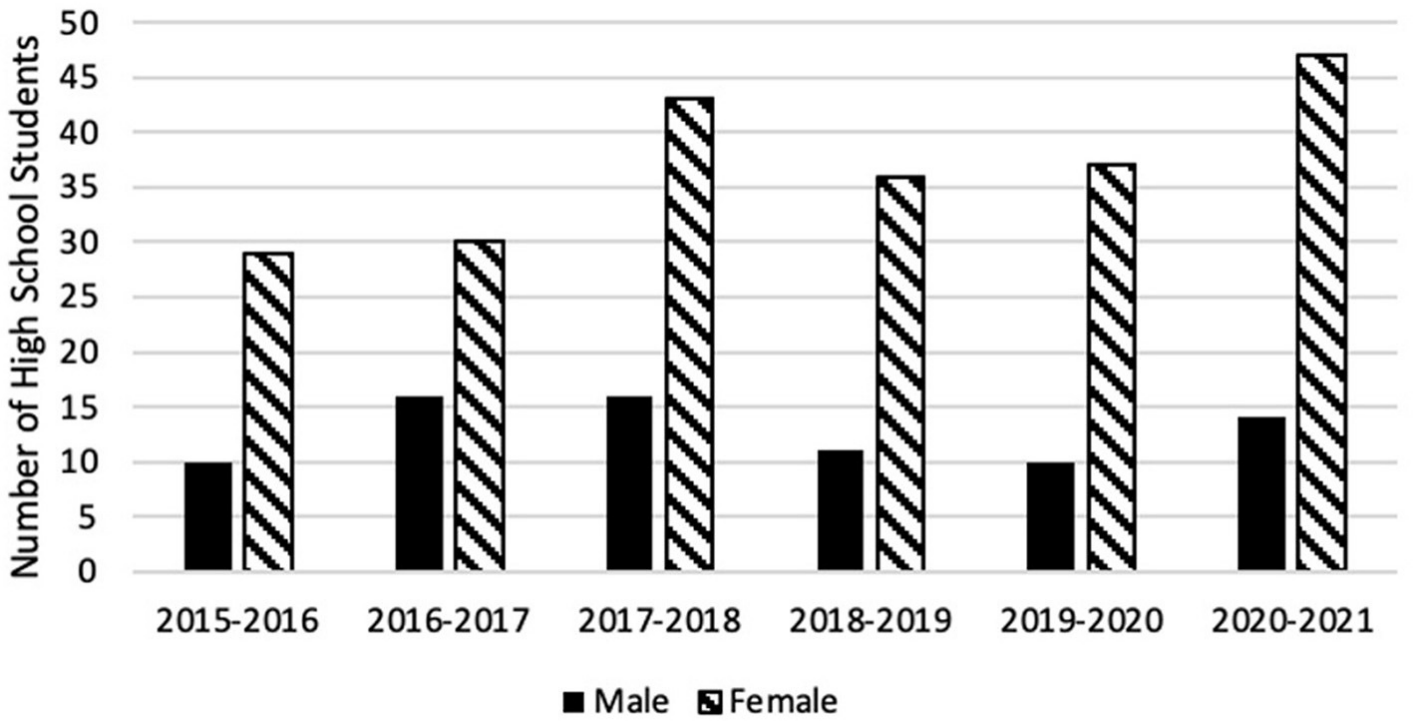
HPREP participant gender by cohort year.

**Figure 2:**
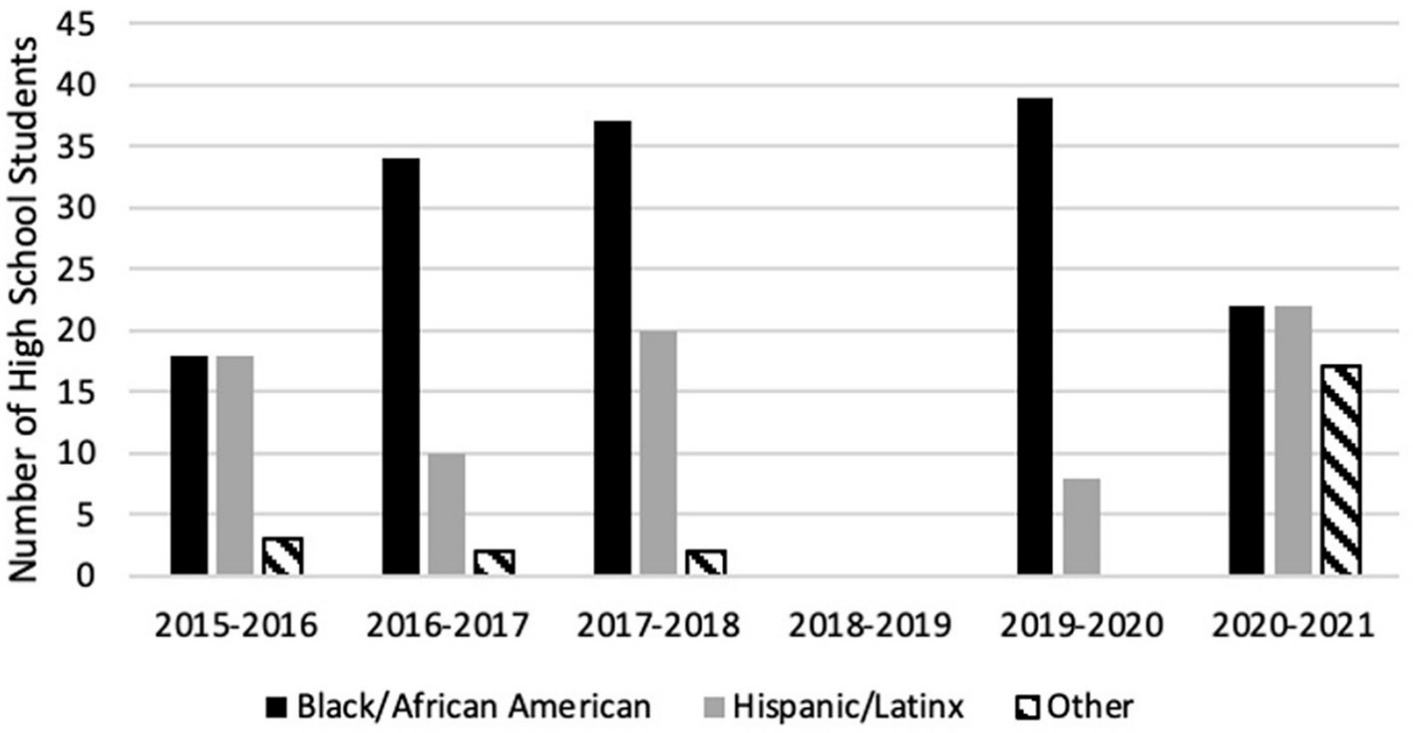
HPREP participant racial/ethnic breakdown by cohort year. Racial and ethnic demographic data from 2018 to 2019 cohort year is missing.

**Figure 3:**
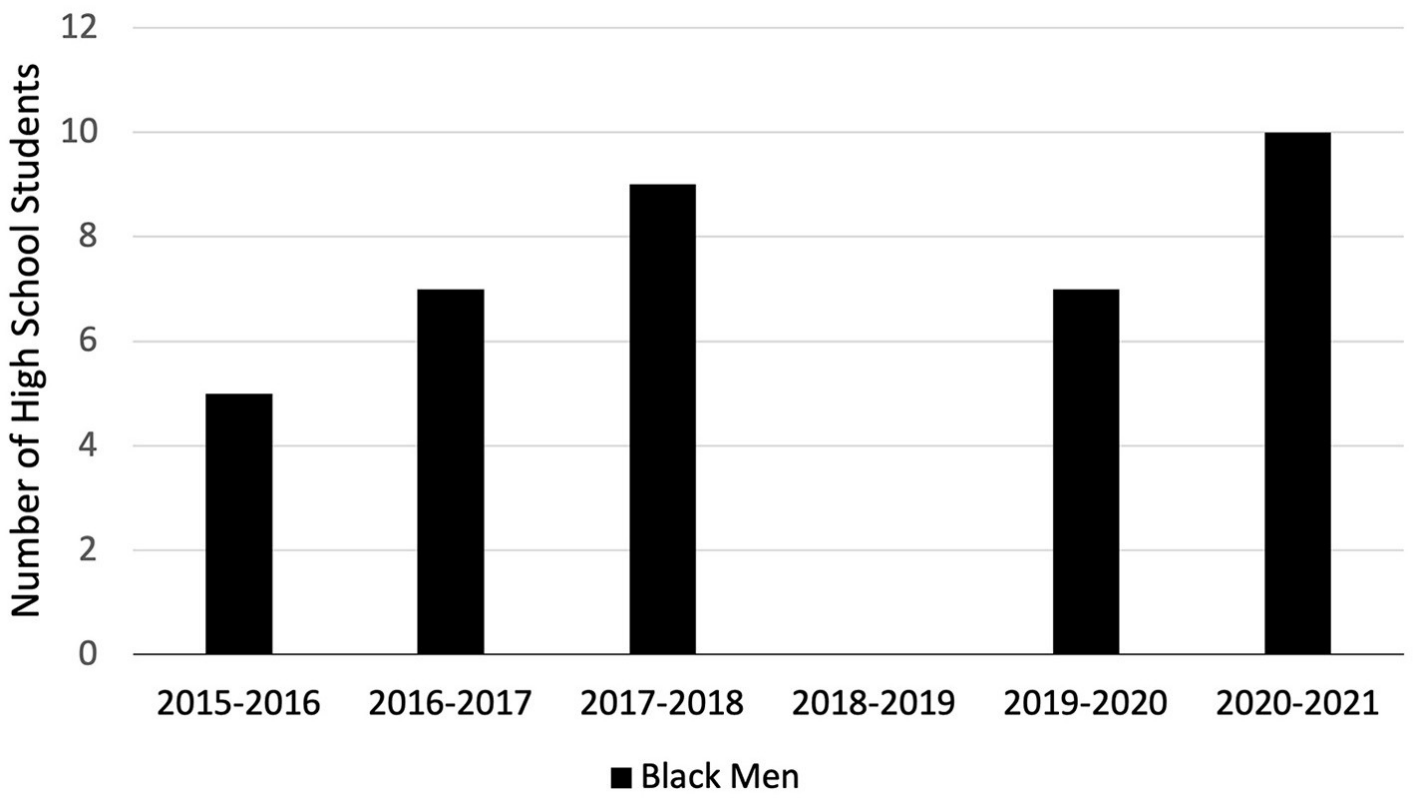
HPREP black male participants by year. Racial and ethnic demographic data from the 2018–2019 cohort year is missing.

Participants ranged in age from 12 to 18 years (mean = 16, SD = 1) with 74% (*n* = 222) being female students (Figure 1). Of 252 respondents, 58% (*n* = 147) identified as Black or African American, 31% (*n* = 77) identified as being Latinx or of Hispanic origin, and 10% (*n* = 24) identified as another race or as bi-racial (Figure 2). Ten or fewer black male students participated in the program every year, including the 2020–2021 year in which 61 total students participated (Figure 3).

The impact of these sessions on students is evaluated through post-program evaluation surveys. After the 2020–2021 program, students felt more comfortable reaching out to an adult for career advice (100%), understanding (97%) and combating (81%) healthcare disparities, and having someone they consider to be a mentor (81%) (24). During the health professions session that took place in the 2020–2021 year, one student remarked: “The most important thing I learned in this session is that you can have a nice life even with all of the stress from medical school. No one actually said this but just from the smiles on everyone's face made me happy.” During the college readiness session, another student commented: “The most important thing I learned from this session was advice on how to stay motivated on days you feel unmotivated. You just have to keep striving for those small goals to get to your big goal and it is okay to remind yourself once in a while why you are working hard.” These comments demonstrate how impactful the program is on students directly after the program; however, the exposure to diverse professions, skills, and knowledge remains the primary barometer of program success. Especially given the relatively young age of the UChicago HPREP students, we hope that their experience will fuel them as they strive to enter the health professions and serve underrepresented populations.

## 4 Discussion

Estimating the implications of these five cohorts is challenging, but there is an established precedent for success from program alumni. Dr. Abdullah Pratt, an emergency medicine physician at the University of Chicago Medical Center, is a graduate of HPREP and the University of Chicago Pritzker School of Medicine. He has been instrumental in creating and maintaining several health-related programs for Chicago youth and was instrumental in gaining support for the University of Chicago's Trauma Center (25, 26). Dr. Pratt describes his experience in the program as an integral stepping stone in his desire to become a doctor as it informs his current practice and community engagement efforts. His mentor in HPREP was Dr. Edward McDonald IV, a then Northwestern University Feinberg School of Medicine student and current gastroenterologist at the University of Chicago Medical Center. In addition to his duties as a physician for gastrointestinal disease, Dr. McDonald channels his knowledge and passion for nutrition into the community. After performing a fellowship in clinical nutrition and receiving culinary training, he created a website and children's book aimed at improving the health through nutrition education (27, 28).

### 4.1 Lessons learned

Based on the University of Chicago HPREP's experiences, the team has learned how to better cultivate future healthcare leaders within its community. Programs that aim to replicate this example may benefit from considering the following lessons.

#### 4.1.1 Developing partnerships and involving your community in your vision

Prior to the COVID pandemic, this chapter of HPREP focused primarily on its students. The post-pandemic era has been a time to recognize those who have been constant supporters and may not know it. Program leadership are building partnerships with businesses that consistently support the program. Showcasing their dedication to increasing underrepresented youth in healthcare to the community may also encourage other businesses to follow their lead.

Developing partnerships with other pathway programs is another potential avenue to create a larger and more unified community. After reaching out to many chapters, program leadership connected with and learned from the chapter at Harvard University, and shared successful practices. DNA extraction experiments that could be performed at home were acquired after learning from their chapter. There has also been increased interest in expanding and hosting events in collaboration with other Chicago chapters. Given the shared passion to support underrepresented youth in healthcare, teamwork will be paramount in taking HPREP beyond individual institutions and toward the city and state levels.

#### 4.1.2 Setbacks to successes, and the importance of reflection

While COVID-19 hampered traditional HPREP activities, it was also the catalyst for positive change. Virtual recruitment allowed efforts to expand to the greater Chicagoland area and some nearby underserved areas in Illinois and Indiana. The 20% increase in participation seen in the 2021 cohort compared to the 2020 cohort was likely due to increased funding and reduced need for transportation.

Beyond acute programming changes, the pandemic provided ample time to be reflective about progress. Given revolving leadership within the program, it is difficult to identify success beyond annual program completion and participant satisfaction. The post-pandemic leadership has facilitated a shift toward addressing trends discovered over multiple cohorts instead of reacting to feedback from only the previous year. Currently, the University of Chicago HPREP chapter is more equipped to identify specific goals and foster targeted solutions to achieve them.

### 4.2 Strengths, limitations, and recommendations

This is the first published longitudinal data aimed at understanding enrollment from a pipeline program for URM students. We found a striking and near-consistent 3:1 enrollment ratio of underrepresented women in medicine compared to male counterparts in our Chicago-based program. Given the program geography and recruitment strategy, the relative lack of black male enrollment is concerning. This work reiterates that pathway programs may be a root cause of national trends in medicine and underscores their importance going forward (7, 8).

The practical implications that stem from this include enacting intentional and strategic efforts addressing the disparities present within local and global communities, and promoting the people and institutions that are dedicated to them. The University of Chicago HPREP is strengthened by the examples set by its clinicians, primarily the physician alumni. These physicians have helped their communities in unique ways, from advocating for a level 1 trauma center on the south side of Chicago to teaching the community about healthy eating habits through children's books and other media. Seeing people who were born and raised in similar neighborhoods and understand local experience can be impactful for students and help promote sustainability and continuous innovation of the pathway.

This report has several limitations. While we are the first group to provide published longitudinal data from our pathway program, this data does not provide college or graduate school admissions outcomes. Large time periods between program participation and eventual enrollment, lost institutional memory within program leadership, and low survey response rates by cohort members post-program make this data difficult to collect. Program leadership and mentors are primarily physicians and surgeons, so other careers in healthcare are underrepresented and likely undertaught to cohort members each year. Guests from other careers are present, but typically for only one of the six workshops. Enrollment may be a result of the methods used to promote the program, even though the choice to apply is based on individual capacity and decisions (see Supplementary Appendix).

Given the experience and lessons learned from this case study, other pathway programs should consider the following actionable recommendations:

1. Consider the program's evolving goals, reflect on how the program structure reflects them, and make changes accordingly. This includes asking participants why they chose to apply to the program, how they heard about it and implementing recommendations from their feedback. This would have been especially useful in this program to target recruitment efforts toward black men. Pathway programs should at least have pre and post-program evaluations to understand changes in perspectives, skills, and knowledge by the end of the program.

2. Promote longitudinal evaluations by preserving core aspects of the program. This is key to interpret program trends and determine where more progress can be made. For instance, keep qualitative questions about which healthcare fields participants like at the end of the program and maintain those question for years.

3. Programs should build systems made with the goal of sustainability and progress in mind, especially if they have revolving leadership. Descriptive information about admissions, budgets, resources, contacts, schedules, outcomes are important for most programs, but others may be useful too. While it is difficult to obtain outcomes data that “proves” the utility of these programs, one suggestion is to create a “mentor and alumni network” such that alumni can expect to be contacted on a frequent basis, whether for mentorship or career outcome purposes.

4. Programs should maintain individual initiatives, but also establish connections between programs in the same city or region. These collaborations will broaden each team's perspectives, and ultimately enrich participants' experiences.

## 5 Conclusions

HPREP continues to have a large influence in shaping the face of healthcare, especially in Chicago, IL. This report aimed to present insights from the University of Chicago HPREP chapter and describe its impact on the local community. The data suggests significant increases in the number of underrepresented minority women becoming physicians and serving Chicago communities in the next decade. The strengths (and pitfalls) of pathway programs may be root causes of national trends in medicine and special attention should be paid to improve involvement of other groups like black men in medicine. By sharing progress metrics, solutions, lessons learned, and recommendations, this case study helps underscore the potentially vital role of pathway programs in improving the future of health care and provides actionable details encouraging improved pathway programs. Given the importance of diverse medical teams and the impact that programs like HPREP have, pathway programs for underrepresented students in medicine should strategically engage with their local communities to help recruit and educate future health professionals.

## Data availability statement

The original contributions presented in the study are included in the article/Supplementary material, further inquiries can be directed to the corresponding authors.

## Ethics statement

Ethical approval was not required for the study involving humans in accordance with the local legislation and institutional requirements. Written informed consent to participate in this study was not required from the participants or the participants' legal guardians/next of kin in accordance with the national legislation and the institutional requirements. Written informed consent was obtained from the minor(s)' legal guardian/next of kin for the publication of any potentially identifiable images or data included in this article.

## Author contributions

MA: Conceptualization, Formal analysis, Funding acquisition, Investigation, Project administration, Supervision, Writing – original draft, Writing – review & editing. AD: Conceptualization, Data curation, Investigation, Methodology, Resources, Supervision, Visualization, Writing – original draft. DD: Data curation, Methodology, Project administration, Visualization, Writing – original draft. SF: Data curation, Formal analysis, Validation, Writing – original draft. IH: Data curation, Formal analysis, Investigation, Methodology, Resources, Visualization, Writing – original draft. IS: Conceptualization, Funding acquisition, Investigation, Project administration, Resources, Supervision, Visualization, Writing – original draft. SW: Data curation, Investigation, Methodology, Project administration, Supervision, Validation, Visualization, Writing – original draft. AP: Validation, Writing – review & editing, Conceptualization, Funding acquisition, Resources, Supervision.
