# Supplementary material for: Solidifying the minority high school student pathway: evidence from the health professions recruitment and exposure program
Source: Front Public Health. 2024 Jun 5;12:1408859. doi: 10.3389/fpubh.2024.1408859 (PMC11188419; doi:10.3389/fpubh.2024.1408859)
Supplement: Supplementary file 1 [file Data_Sheet_1.PDF]

Frontiers of Public Health Supplementary Material

Table of Contents

- Supplemental Information – AAMC URM Definition & Methods for Recruitment – pg. 2
- Supplemental Figure 1 – Application Rubric – pg. 3
- Supplemental Figure 2 and 3 – Example Schedules – pg. 4
- Supplemental Figure 4 – Program Activities – pg. 6
- Supplemental Figure 5 – Example Post-Workshop Survey – pg. 12
- Supplemental Figure 6 – Example Post-Program Evaluation – pg. 18

Supplemental Information. AAMC URM Definition

Excerpt from AAMC website (linked below)

"Underrepresented in medicine means those racial and ethnic populations that are underrepresented in the medical profession relative to their numbers in the general population."

Adopted by the AAMC's Executive Council on June 26, 2003, the definition helps medical schools accomplish three important objectives:

- a shift in focus from a fixed aggregation of four racial and ethnic groups to a continually evolving underlying reality. The definition accommodates including and removing underrepresented groups on the basis of changing demographics of society and the profession,
- a shift in focus from a national perspective to a regional or local perspective on underrepresentation, and
- stimulate data collection and reporting on the broad range of racial and ethnic self-descriptions.

https://www.aamc.org/what-we-do/equity-diversity-inclusion/underrepresented-in-medicine

Supplemental Information. Methods for Recruitment

Recruitment team members reach out to Chicago public schools that were on previous HPREP participation lists (the sheet curated by past team members). The team personally emails the principal or other listed contact on the sheet.

During the 2020-2021 year, the recruitment team expanded the program to underserved schools outside of our traditional Chicagoland region. This included rural high schools in central Illinois and northwest Indiana.

Previous teams had done site visits to encourage participation.

Figure 1. 2020-2021 Application Rubric

Score	Characteristics
5	1) Essay clearly and fully addressed the prompt.<br>2) Clear and substantial reasoning for wanting to join HPREP (listed at least 2 to 3 examples)<br>3) List two or more concrete extracurricular involvements. (not applicable for freshman)<br>4) Utilized complete sentences and provided fully formed responses.
4	3 of 4 of a 5 point application is achieved
3	2 of 4 of a 5 point application is achieved
2	1 of 4 of a 5 point application is achieved
1	0 of 4 of a 5 point application is achieved

1. Using the rubric above. Grade your assigned applications. Assign each application a score and detail which of the 4 characteristics they exemplified.
2. Enter your grade and reasoning into your specific tab on the spreadsheet named HPREP Application 2020 (Responses)
3. Double check that your scores align with the proper application
4. Each applicants scores will be added up from their two graders
5. The applicant's with the top 50 scores will be accepted. We will also give a slight priority to juniors and seniors.

Figure 2. Example Daily Schedule (example from 2016-2017 Session 1)

Learning goals and objectives:

1A: To gain a better understanding of what it means to be a doctor and the extent of Health Care disparities that are present in the south side

1B: To gain hands-on experience in medically related procedures to gain a better appreciation of the medical field

1C: To stimulate an interest in pursuing a higher level of education and give them the tools and practical steps required to do so

1D: To develop presentation and professional skills and expand students' knowledge of healthcare professions in preparation for objective 3D

**Time**	**Speaker/ Volunteers**	**Description**	**Location**	**Learning Objectives**	**Who is responsible?**
8:30-9:00	HPREP Board	Check-in for students	BSLC Lobby		
9:00-9:45	HPREP Board	Icebreakers, group bonding	BSLC Lobby		
10-10:15		Welcome/Introduction, Provident Foundation	BSLC 109		
10:15-11:00	Dr. Tanksley	My Path to Medicine	BSLC 109	1A	
11:15-12:15	EM Residents	Interactive Session (ultrasound session)	Sim Center, Mitchell 7th floor	1B	
12:15-1:00	Med students	Mentorship Lunch - Introduction and Discussion on Medicine and Science, Healthcare Disparities *(also do pre-study survey)*	BSLC Lobby	1C	
1:00-1:50	Med Students	Medical Student Panel: Pathway to Medicine	BSLC 109	1A, 1C	
2:00-3:00	Medical students	Project intro: choose a topic, how to research, how to cite, Poster making skills	Small groups	1D	

Figure 3. Example Daily Schedule (examples from 2016-2017 Session 4)

Saturday 1/28 (SESSION 4) (Logan Leads)

Health Career Focus: Nutrition and Public Health (addresses post-survey question concerning issues of obesity, nutrition and health complications such as heart disease)

Learning goals and objectives:
4A: To gain an understanding of careers in nutrition
4B: To gain a better understanding of proper nutrition as it relates to their own health
4C: To gain hands-on experience with basic cardiovascular anatomy and to learn of the long-term cardiovascular effects of poor nutrition
4D: To learn how to create a resume that can be used for college and/or job applications

Time	Speaker/ Volunteers	Description	Location	Learning Objectives	Who is responsible?
9:00-9:50	Valerie Reynolds	Nutritionist Career Overview (reference public health implications)<br>*path to RD	BSLC 109	4A	
10:00-10:50		Interactive Session: Nutrition Case Workshop	Histo Rooms	4B	
11:00-12:30	Surgery Interest Group	Interactive Session: Sheep Heart Dissection	Histo Rooms	4C	
12:30-1:15	Med Students	Mentorship Lunch - Visualization and Goal Setting	BSLC Lobby	1C	
1:20-1:40	Booth Volunteers, Med Students	Lecture II: Now that students have identified where they want to go, the next step is to prepare a resume for consideration by the school/employer that shows that they are the right candidate.	BSLC 109	4D, 1C	
1:50-3:00	Booth volunteers, Med students	Activity II: Students are given a sample resume and work together to format their activities and experiences into a resume	Computer labs	4D, 1C	

Figure 4. Program Activities

Interactive Activity	Competency Domain	Skills Learned & Additional information
Case Discussions	Clinical Sciences of Medical Knowledge	Examples of previous cases include high blood pressure with no health insurance, substance misuse and abuse, and nutrition-related.
Contacting Mentors Practice	Personal Development	Have previously a list with names, gender, contact information, and interests of medical students. Ask students to identify a potential connection and provide guidance on how to ask for mentorship.
DNA Extraction Experiments	Basic Sciences of Medical Knowledge, Clinical Sciences of Medical Knowledge	Led by an MD-PhD candidate. Students consider how knowledge of the human body is applied to medical tests for genetic diseases and cancers, as well as forensics.
Ice-Breakers and Energizers	Personal Development, Communication	These activities encourage teamwork and a sense of community within the cohort.

Longitudinal Research Project	Research, Communication	Small groups of 4-7 students select a topic with the help of their group mentor(s). They use internet searches to research the topic every week to learn about the health problem, the stakeholders involved, the medical basis of the issue, proposed solutions (if possible), and community resources to help alleviate the issue in their communities. They produce posters as a visual aid of their work. On the final day, they present their project to their peers and families, who are invited to support.
Mock Interviews	Personal Development, Communication	Typically done in pairs with the help of experts. Booth School of Business volunteers have helped with this in the past.
Personal Statement and Resume Writing Workshops	Personal Development, Communication	This session provides practical knowledge that many if not all of the students will use in the near future. College and medical school admissions teams, as well as Booth School of Business volunteers, have helped with this in the past to provide key insight in how to succeed in these tasks.

Physical Therapy Exercises	Clinical Skills	Physical Therapy bands are purchased to supplement this session. Student keep these materials. Session is led by physical therapists, who share exercises that students can try and teach others, why they are used, etc.
Sheep Heart Dissection	Clinical Sciences of Medical Knowledge, Clinical Skills	This activity helps student see anatomy in real life. Students get experience using medical tools to perform simple operations. This is typically led by surgical faculty or residents, but can be led by medical students.
Simulation Center Activities (live ultrasound scan, peripheral IV ultrasound scan, CPR sessions)	Clinical Skills, Clinical Sciences of Medical Knowledge, Communication	The simulations aim to expose students to a spectrum of tasks that healthcare professionals perform. Where possible, they learn about the anatomy and physiology behind the tests. Students also communicate to the instructors and their peers throughout these exercises.

'Stop the Bleed' Training	Clinical Skills	This session helps teach students first aid skills that may be useful in emergency situations, as well as general medical knowledge. Typically led by Dr. Abdullah Pratt with assistance from Emergency Medicine residents. Materials for this are provided by the Emergency Medicine department. For more information, see: https://civicengagement.uchicago.edu/news/training-the-next-generation-to-tackle-health-disparities-and-community-violence
Suture and Knot Tying Practice	Clinical Skills	The primary goal of this session is to expose students to basic surgical principles and help them realize that the skills necessary to suture are attainable for them. Expired suture is provided by the University of Chicago Department of Surgery. HPREP leadership provides suture pads and tools. Either surgical attendings, residents the medical student surgery interest group, or clinical medical students led the sessions.
Tours of Medical campus		Goal is to expose students to the medical setting and what it can look like in person.

Vital Sign and Physical Exam (Heart and Lung) Practice	Clinical Sciences of Medical Knowledge, Clinical Skills	This activity provides exposure to key clinical tests and measures that are applicable to many careers in healthcare. Where possible, students are taught about basic anatomy and physiology that underlies the tests and maneuvers. Medical student mentors lead these sessions.
**Pedagogy Activity**		
"My Path to" Talks	Personal Development	These are typically performed by various professionals in healthcare
Lectures on Topics	Clinical Sciences of Medical Knowledge	Examples of topics include Adolescent Health (by pediatrics residents) and Diabetes Mellitus (by an endocrinologist)
Panels	Personal Development	Previous panels examples include a medical student panel and a care team panel (nurse, pharmacist, resident, attending physician, etc.)

Competency Domains are https://my.clevelandclinic.org/-/scassets/files/org/lerner-college-medicine/portfolio-approach-to-competency-based.pdf?la=en#:~:text=To%20graduate%2C%20a%20student%20must,personal%20development%2C%20and%20reflective%20practice.

Figure 5. Example Post-Workshop Survey (from 2019-2020 Session 2)

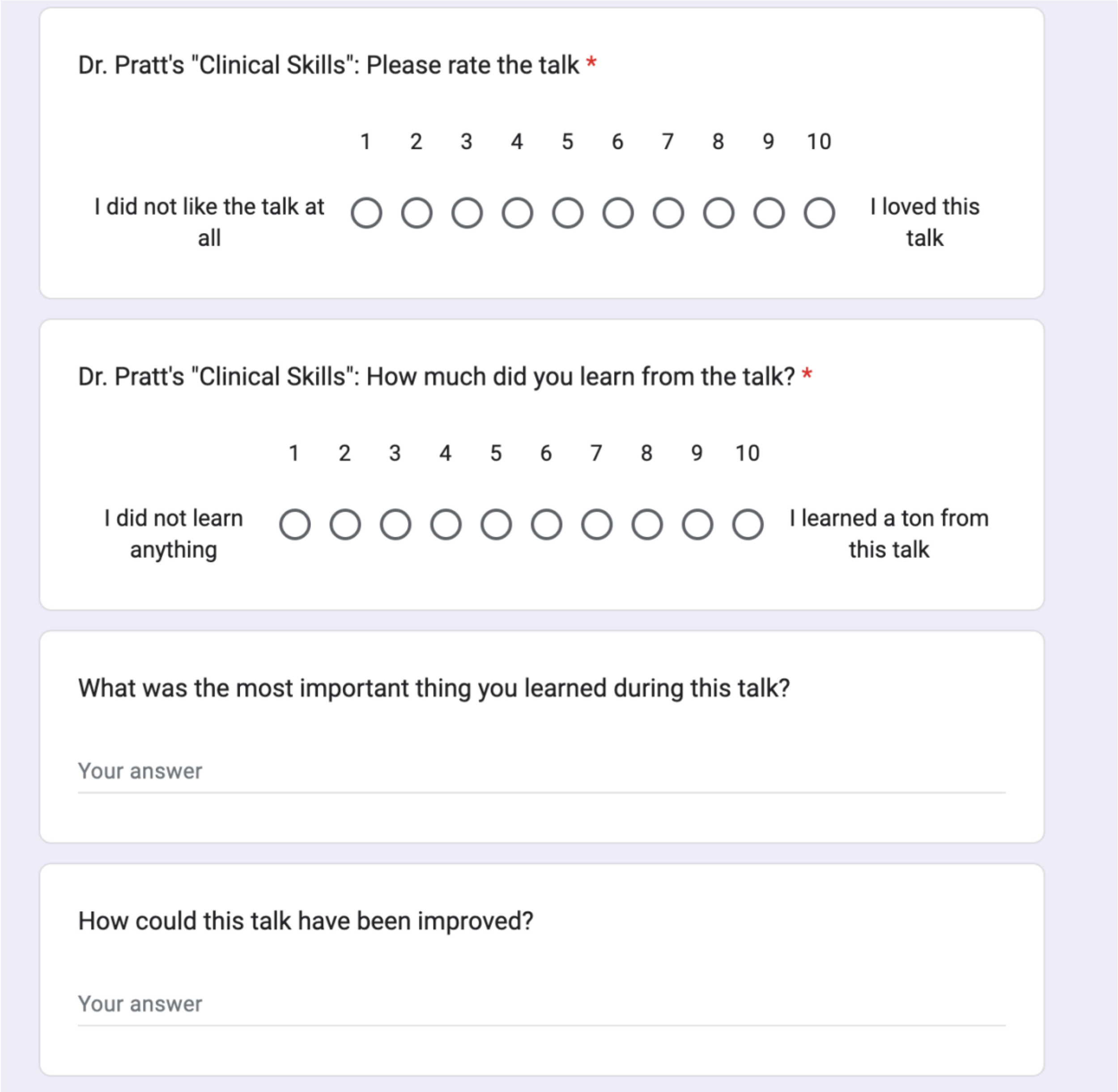

Dr. Pratt's "Clinical Skills": Please rate the talk *

	1	2	3	4	5	6	7	8	9	10	
I did not like the talk at all	○	○	○	○	○	○	○	○	○	○	I loved this talk

Dr. Pratt's "Clinical Skills": How much did you learn from the talk? *

	1	2	3	4	5	6	7	8	9	10	
I did not learn anything	○	○	○	○	○	○	○	○	○	○	I learned a ton from this talk

What was the most important thing you learned during this talk?

Your answer

How could this talk have been improved?

Your answer

Clinical Skills HPI: Please rate the sessions overall *

	1	2	3	4	5	6	7	8	9	10	
I did not like the HPI session	○	○	○	○	○	○	○	○	○	○	I loved the HPI session

Clinical Skills HPI: How much did you learn from this session? *

	1	2	3	4	5	6	7	8	9	10	
I did not learn anything during the HPI session	○	○	○	○	○	○	○	○	○	○	I learned a ton during the HPI session

Stop the Bleed: Please rate the sessions overall *

	1	2	3	4	5	6	7	8	9	10	
I did not like the injection session	○	○	○	○	○	○	○	○	○	○	I loved the injection session

Stop the Bleed: How much did you learn from this session? *

	1	2	3	4	5	6	7	8	9	10	
I did not learn anything during the injection	○	○	○	○	○	○	○	○	○	○	I learned a ton during the injection session

Sheep Heart Dissection: Please rate the sessions overall *

	1	2	3	4	5	6	7	8	9	10	
I did not like the blood pressure session	○	○	○	○	○	○	○	○	○	○	I loved the blood pressure session

Sheep Heart Dissection: How much did you learn from this session? *

	1	2	3	4	5	6	7	8	9	10	
I did not learn anything during the blood pressure session	○	○	○	○	○	○	○	○	○	○	I learned a ton during the blood pressure session

Favorite clinical skills session *

- ○ HPI (History of Present Illness)
- ○ Stop the Bleed
- ○ Sheep Heart Dissection

Why?

Your answer ______________________________

Least favorite clinical skills session *

- ○ HPI (History of Present Illness)
- ○ Stop the Bleed
- ○ Sheep Heart Dissection

Why?

Your answer

Ethical Dilemma Workshop: Please rate the session *

	1	2	3	4	5	6	7	8	9	10	
I did not like the session at all	○	○	○	○	○	○	○	○	○	○	I loved this session

Ethical Dilemma Workshop: How much did you learn from this session? *

	1	2	3	4	5	6	7	8	9	10	
I did not learn anything	○	○	○	○	○	○	○	○	○	○	I learned a ton from this session

What was the most important thing you learned during this session?

Your answer

How could this session have been improved?

Your answer

I enjoyed session 2 of HPREP *

- ○ Strongly Agree
- ○ Agree
- ○ Neutral
- ○ Disagree
- ○ Strongly Disagree

I gained new knowledge during session 2 of HPREP *

- ○ Strongly Agree
- ○ Agree
- ○ Neutral
- ○ Disagree
- ○ Strongly Disagree

Submit

Clear form

<u>Figure 6. 2020-2021 HPREP Post-Program Evaluation</u>

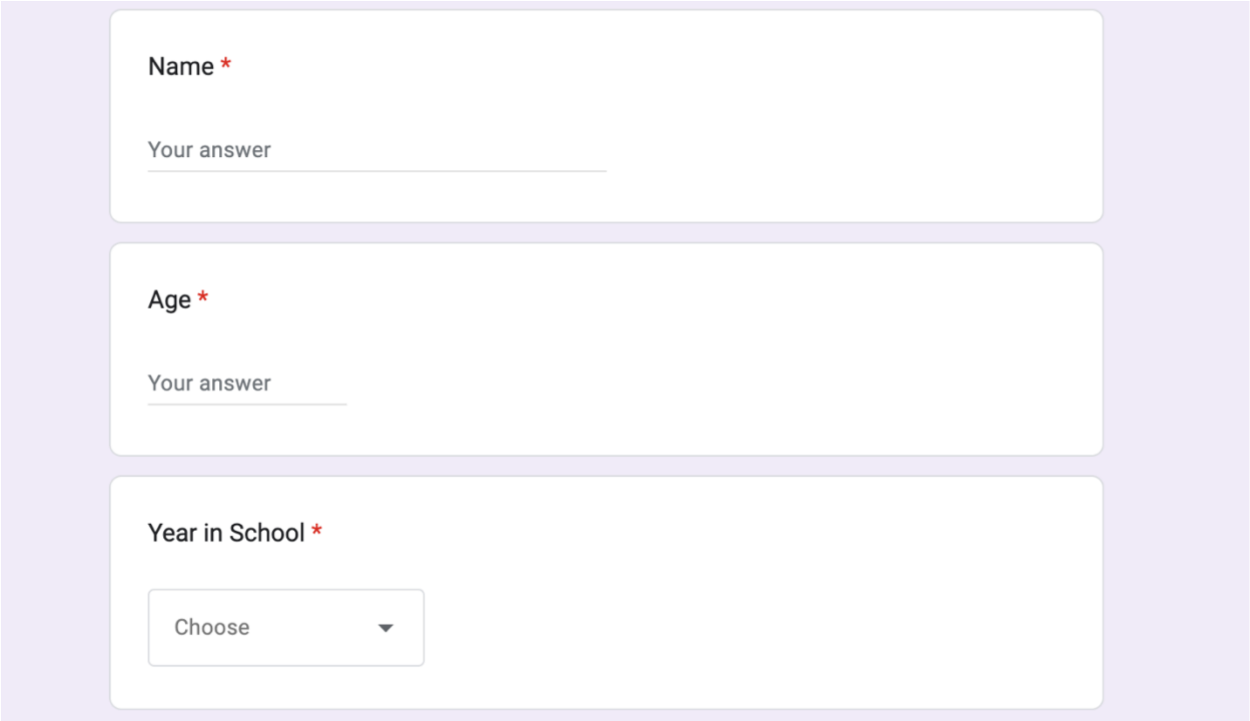

Name *

Your answer

Age *

Your answer

Year in School *

Choose

What is your gender? *

- ○ Female
- ○ Male
- ○ Non-Binary
- ○ Prefer not to say
- ○ Other: ______

What is your race/ethnicity? *

- ☐ Black or African American
- ☐ Hispanic or Latinx
- ☐ White
- ☐ Asian
- ☐ American Indian and Alaska Native
- ☐ Other: ______

What Chicago neighborhood are you from? *

Choose

What type of school do you attend? *

Choose

Do you have a family member that works in healthcare? *

Choose

If so, what do they do?

Your answer

How confident are you that you want a career in healthcare? *

	1	2	3	4	5	6	7	8	9	10	
Not at all confident	○	○	○	○	○	○	○	○	○	○	I definitely want a career in healthcare

Do you have a specific career in mind? *

- ○ Yes
- ○ No

If so, what career would you like to pursue?

Your answer

Please check all the professions you have heard about *

- ☐ Doctor
- ☐ Nurse
- ☐ Advanced Practice Nurse (APN)
- ☐ Physician Assistant (PA)
- ☐ Genetic Counselor
- ☐ Registered Nutritionist
- ☐ Pharmacist
- ☐ Speech Pathologist

Please check all the professions whose jobs you would be able to explain to others *

- [ ] Doctor
- [ ] Nurse
- [ ] Advanced Practice Nurse (APN)
- [ ] Physician Assistant (PA)
- [ ] Genetic Counselor
- [ ] Registered Nutritionist
- [ ] Pharmacist
- [ ] Speech Pathologist

Do you currently have someone you consider to be a mentor? *

- ○ Yes
- ○ No

How valuable is mentorship in healthcare careers? *

	1	2	3	4	5	6	7	8	9	10	
Not at all valuable	○	○	○	○	○	○	○	○	○	○	Extremely valuable

How comfortable would you be reaching out to an adult (health care professional, med student, etc) for career advice? *

	1	2	3	4	5	6	7	8	9	10	
Not at all comfortable	○	○	○	○	○	○	○	○	○	○	Extremely comfortable

How much do you know about health disparities? *

	1	2	3	4	5	6	7	8	9	10	
I don't know anything about health disparities	○	○	○	○	○	○	○	○	○	○	I am an expert on health disparities

I know what can be done to reduce health disparities *

- ○ Strongly Agree
- ○ Agree
- ○ Neutral
- ○ Disagree
- ○ Strongly Disagree

I can define the term "health disparities" *

Choose ▾

Define "health disparity" *

Your answer

Dr. Tanksley "Health Inequities ": Please rate this talk *

	1	2	3	4	5	6	7	8	9	10	
I did not like this talk at all	○	○	○	○	○	○	○	○	○	○	I love this talk

Dr. Tanksley "Health Inequities ": How much did you learn from this talk? *

	1	2	3	4	5	6	7	8	9	10	
I did not learn anything	○	○	○	○	○	○	○	○	○	○	I learned a ton from this talk

What was the most important thing you learned during this talk?

Your answer

How could this talk have been improved?

Your answer

We would like to provide all HPREP participants with a resource packet at the end of the program. What information would you want provided in a resource packet?

Your answer

What was your favorite HPREP activity?

Your answer

What was your least favorite HPREP activity?

Your answer

What should we add or improve for next year's program?

Your answer

Submit

Clear form
